# Application of Rasch analysis to the parent adherence report questionnaire in juvenile idiopathic arthritis

**DOI:** 10.1186/s12969-016-0105-5

**Published:** 2016-07-28

**Authors:** Karine Toupin April, Johanne Higgins, Debbie Ehrmann Feldman

**Affiliations:** 1 Children’s Hospital of Eastern Ontario Research Institute, 401 Smyth Road, room R1132, Ottawa, Ontario Canada; 2 Department of Pediatrics, University of Ottawa, Ontario, Canada; 3 École de Réadaptation, Université de Montréal, and researcher, Centre de recherche interdisciplinaire en réadaptation (CRIR) - Institut de Réadaptation Gingras-Lindsay-de Montréal du CIUSS-Centre-Sud de Montréal, Montreal, Canada; 4 École de Réadaptation and Institut de recherche en santé publique de l’Université de Montréal (IRSPUM), Université de Montréal, Centre de recherche interdisciplinaire en réadaptation (CRIR), Montreal, Canada

**Keywords:** Juvenile arthritis, Patient compliance, Questionnaires, Psychometrics

## Abstract

**Background:**

Adherence to treatment in children with juvenile idiopathic arthritis (JIA) is associated with better outcomes. Assessing patient adherence in JIA, as well as attitudes and beliefs about prescribed treatments, is important for the clinician in order to optimize patient management. The objective of the current study was to evaluate the psychometric properties of the Parent (proxy-report) Adherence Report Questionnaires (PARQ), which assesses beliefs and behaviors related to adherence to treatments prescribed for JIA.

**Methods:**

A Rasch analysis was conducted on data collected with parents of children with JIA from two studies in which the PARQ was used as a measure of adherence.

**Results:**

The PARQ showed preliminary evidence of multidimensionality with two factors, accounting for 38 % and 27 % of the variance respectively. The PARQ in its original version does not adhere to expectations of the Rasch model. A transformed version of the PARQ obtained by deletion of the general adherence scale and modification of visual analog scales into 5-point likert scales improved fit to the model and showed preliminary evidence of unidimensionality.

**Conclusions:**

The PARQ was transformed based on the results of the Rasch analysis. The transformed version of the PARQ shows preliminary evidence of unidimensionality and may allow computation of a total score, although further testing is needed to verify these findings.

## Background

Higher levels of adherence are associated with better health outcomes among children with chronic diseases [1] including juvenile idiopathic arthritis (JIA) [2, 3]. Valid, reliable, and easy to administer measures assessing patient adherence, as well as factors associated with adherence to prescribed treatments, must be available for use in clinical practice in order to optimize patient management [1]. In JIA, adherence may involve gaining access to the medications, taking the right dosage at the appropriate times, as well as performing prescribed exercises and wearing splints according to the instructions of their therapist. Although there is no gold standard to assess adherence [1], using patient self-report questionnaires is the most common and practical way to evaluate adherence [1]. A few questionnaires have been developed to assess adherence in pediatric chronic diseases such as JIA but none assesses adherence to the various modalities used in JIA in a valid manner [1].

Our research team developed the Parent Adherence Report Questionnaire (PARQ) aimed at measuring patient and parent beliefs and behaviors related to adherence to various treatments prescribed for JIA in order to elucidate adherence issues and act upon them [4, 5]. The PARQ has shown satisfactory construct validity and test-retest reliability using Classical Test Theory [4]. However, the assumption of unidimensionality, imperative for construct validation and proof of interval-level measurement permitting the summation of individual items into a total score, have not been verified. Also, other important psychometric properties such as the usefulness of the response categories for each item and differential item function (item bias) have not been investigated. At this time, the interpretation of the PARQ remains mostly qualitative, as no overall score can be derived from the individual items. Rasch analysis, a modern model for evaluating psychometric properties of self-report measures, can provide this valuable information and informs on how well the items contribute to defining the construct of the questionnaire: adherence to treatment in children [6]. The aim of the present study is to evaluate the PARQ using Rasch analysis.

## Methods

### Instruments

The development of the PARQ was guided by the World Health Organization (WHO) conceptual framework [7], as well as a literature review of studies on adherence in general pediatrics and JIA [4]. The WHO model is comprised of five dimensions of factors associated with adherence: 1) social and economic factors, 2) health care team and system-related factors, 3) condition-related factors, 4) therapy-related factors, and 5) patient-related factors [7]. Several consultations with a team of pediatric rheumatology health professionals were performed to identify elements that should be included in the questionnaire [4]. Once the PARQ was developed, this team was consulted to determine its face and content validity, and its feasibility [4].

The PARQ was tested for construct validity and test-retest reliability, which were shown to be satisfactory when medication and exercise scores were compared with the General Adherence Scale and to diary reports of medication and exercise-related behaviors [4]. The PARQ was pilot tested for ease of use in five English-speaking caregivers of youth with JIA, and translated into French by a bilingual professional translator [4]. The PARQ has since been used in various research projects [3, 5, 8] and a child-report version (i.e.*,* Child Adherence Report Questionnaire (CARQ)) has been developed and pilot-tested among youth with JIA [3, 5].

The PARQ focuses on the domain of patient-related factors (e.g.*,* knowledge, attitudes, beliefs, perceptions, and expectations) of the WHO model. It assesses which member of the family is responsible for making sure that the child adheres to treatment. The following items are scored on a 100 mm visual analog scale (VAS): frequency with which children follow their prescribed treatments (medication, exercise regimen and splints), difficulties experienced in following the various forms of treatment, the frequency of negative reactions associated with following the various forms of treatment, and the degree to which the treatments are perceived as helpful. The Morisky scale, an index addressing barriers to medication adherence [9] is also included in the PARQ. The Morisky scale is comprised of four yes/no questions related to forgetting to take medication, neglecting to take medication, stopping the medication when the child felt better or worse than before, and choosing a type of medication (4-point scale). Finally, parent and child treatment preferences and perceptions regarding treatment helpfulness and also dissatisfaction with care are included as these may influence adherence.

### Procedure

Rasch analyses were performed on data collected from two studies conducted in Vancouver and Montreal (Canada) in which the PARQ was used as a measure of adherence. The first study was a longitudinal survey assessing the adherence to treatment and its associated factors (e.g.*,* child’s disease severity, child’s age, caregivers’ perceived helpfulness of treatments, use of complementary and alternative health care) in a sample of parents of 180 children with JIA [2, 8]. The second was a cross-sectional survey of 55 children with JIA and their parents to compare their respective perceptions of treatment adherence and quality of life and to determine the association between adherence and health outcomes [5]. In both studies, we also assessed health related quality of life (HRQOL) using the Juvenile Arthritis Quality of Life Questionnaire (JAQQ) and extracted information from medical charts on disease severity (using the active joint count, representing the number of joints with active inflammation as rated by the rheumatologist) and disease duration. Thus, from the two studies, we had a total of 235 PARQ baseline questionnaires for this study.

### Analysis

First, a principal component analysis (PCA) was performed to determine the factor structure and the dimensionality of the PARQ questionnaire using SAS (version 9.0). Rasch analyses were then conducted to assess how well the data fit the Rasch partial credit model. Content validity, construct validity, as well as other psychometric properties, such as the appropriateness of response categories, floor and ceiling effects, item bias and reliability were evaluated. The RUMM 2030 [10] computer software program was used for Rasch analyses [11].

#### Principal component analysis

We analyzed the PARQ baseline data to perform a preliminary examination of its dimensionality and factor structure using PCA [12].

#### Rasch analyses

**Fig. 1:**
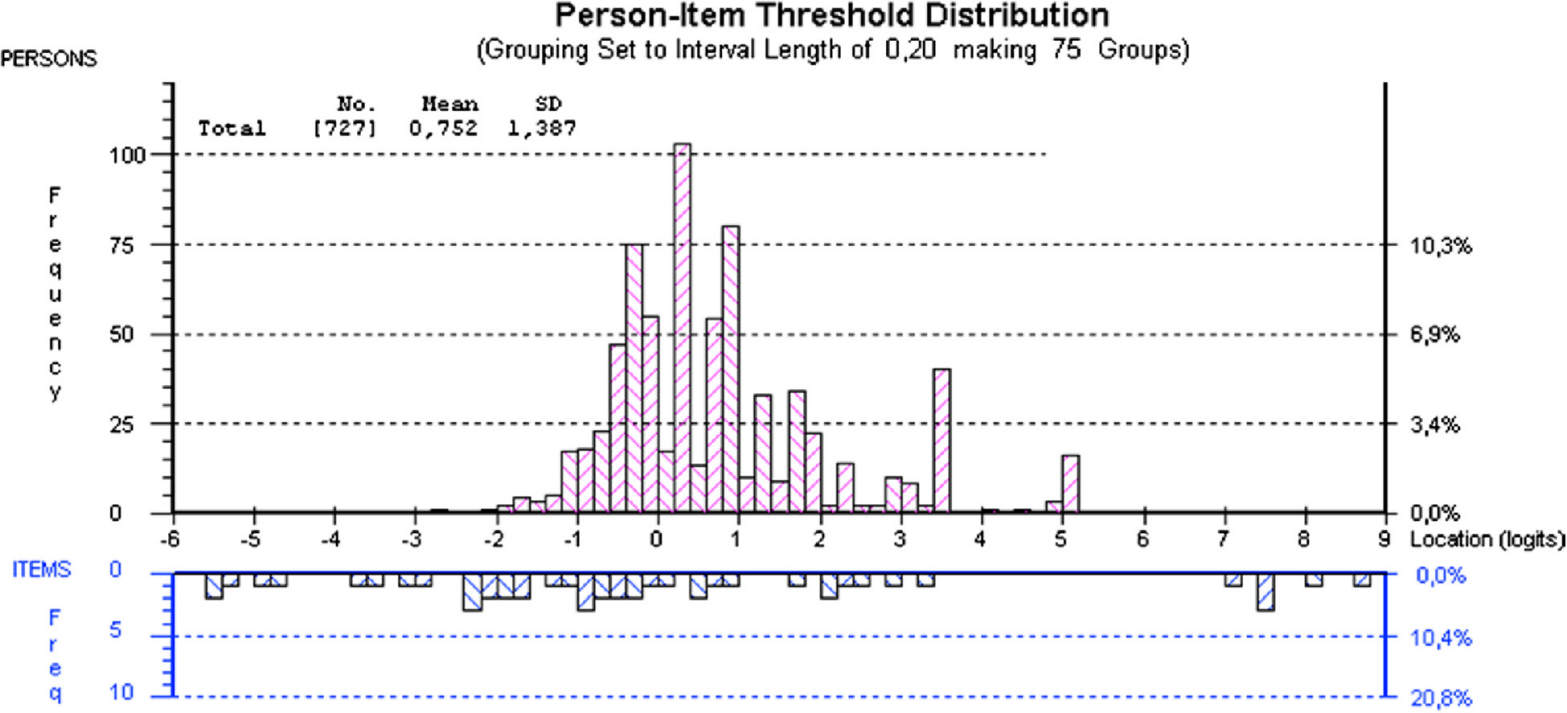
Person-item threshold distribution. Figure 1 shows the distributions of persons (*top*) and items (*bottom*) for the transformed PARQ. The item thresholds spread from approximately −10 to 13 logits, which is adequate

Baseline data were analyzed to evaluate their fit to the Rasch partial credit model [13]. This model is used when items within a test or index are scored on different scales. Indeed, the PARQ items are scored on a dichotomous scale (0–1), an ordinal scale (0 to 4) and a VAS (0 to 100 mm). The Rasch model describes the relationship between an item and a person’s response to this item and is useful in validating measures. When items *meet the expectations of* the Rasch model, they are said to ‘fit’ the model and they are placed in order of difficulty along a ruler or scale that represents the trait under study, which is adherence in this case. On this ‘item-person map’, the bars represent the distribution of items according to their difficulty and the distribution of persons according to their ability to adhere to treatment. Easy items are located towards the left of the graph while difficult items are at the right. Likewise, persons who exhibit lower adherence to treatment are located towards the left and those exhibiting higher levels of adherence are located towards the right (see Fig. 1). In the case of the PARQ questionnaire, difficult items are items for which achieving adherence is harder to attain and the ability of a person represents their level of adherence to a particular treatment.

Ideally, for items to “fit” the model and for the questionnaire to have adequate content validity, items should be spread evenly on the continuum of difficulty level and have a wide range (from at least −3 to +3 logits) (see Fig. 1).

Construct validity is attained when persons and items have adequate fit statistics (e.g.*,* item and person standardized fit residuals between ±2.5 with a mean of 0 and non-significant chi-squares and F statistics). These methods and their criteria are fully described elsewhere [14–17]. Construct validity is also attained when none of the items display differential item functioning (DIF) or item bias [18]. Items are considered biased if they change their level of difficulty depending on the group of persons being assessed (e.g.*,* boy vs. girl), which violates the expectations of the Rasch model. DIF was deemed to be present if analyses of variance were significant (Bonferroni-corrected p value of 0.001389).

The usefulness of the response categories was assessed for adequacy of their response options. For polytomous items, responses should be adequately distributed across the response categories, and this is usually indicated as a minimum of 10 observations in each rating scale category [19]. The reliability of the questionnaire was assessed by the person separation index, which is interpreted as a Cronbach’s α [20]. It indicates how well the items discriminate persons into different ability levels.

## Results

### Participants’ characteristics

Characteristics of the participants included in the analyses are shown in Table 1. The distribution of JIA was as follows: polyarthritis (23.61 %), oligoarthritis (36.48 %), enthesitis-related arthritis (11.16 %), systemic arthritis (9.44 %), psoriatic arthritis (10.30 %) or another type of arthritis (9.01 %). In terms of treatments, 76.09 % of children were prescribed medication (mostly NSAIDs (54.89 %), non-biologic DMARDs (45.53 %) or TNF alpha inhibitors (6.38 %), corticosteroids (8.94 %)), 68.85 % were given exercises and 15.43 % were prescribed splints. Mean scores on 100 mm VAS for the parent-reported adherence to medication, exercises and splints were 86.14 mm (SD = 25.98 mm), 54.52 mm (SD = 31.65 mm) and 49.18 mm (SD = 41.21 mm) respectively. Mean scores for the parent-reported difficulty in following medication, exercises and splints were 20.51 mm (SD = 25.93 mm), 35.99 mm (SD = 30.11 mm) and 34.89 mm (SD = 37.29 mm) respectively. Medication was felt to be the most helpful (mean score = 83.30 mm, SD = 24.74 mm) compared to exercises (mean score = 67.64 mm, SD = 29.95 mm) and splints (mean score = 65.07 mm, SD = 35.56 mm).

**Table 1:** Demographic and disease related characteristics of the families included in the analyses for the PARQ

Characteristics	PARQ data
	*n* = 235 parents of children with JIA^a^
Children’s sex, female, n (%)	169 (71.91)
Children’s age, years, mean (SD)	10.80 (4.19)
Disease duration, years, mean (SD)	4.66 (3.79)
Disease severity, AJC, mean (SD)	1.62 (3.37)
HRQOL, mean (SD)^b^	2.20 (1.12)
Pain, mean (SD)^c^	17.18 (22.81)

*SD* standard deviation, *AJC* active joint count

^a^The sample size includes families involved in the two studies: families of 180 children and 55 children

^b^On a scale from 1 to 7, 7 being a worse HRQOL

^c^On a visual analog scale from 0 to 100, 100 being worse pain

#### Principal component analyses

While the type of data for some of the items (ordinal) along with missing data precluded definite conclusions about the factor structure revealed by the PCA, it helped us identify and understand the number of dimensions within the construct and to identify items unrelated to a one-dimensional concept of adherence. The PCA indicated the presence of two main factors that explained 38 and 27 % of the variance respectively. Most of the 17 items loaded on the first factor. However, three items did not load on the first factor: Forgetting to take medication during the last 3 months, neglecting to take medication during the last 3 months and difficulty in doing prescribed exercises. These items appear to form a second factor and may contribute to the multi-dimensionality of the scale. All items were retained for the Rasch analyses of the PARQ questionnaire.

#### Rasch analyses

The fit of the baseline data when all 17 items of the PARQ were considered produced a significant item-trait interaction (chi-square = 244.76; *p* < 0.05). This is an indication that the data did not fit the Rasch model. All items originally scored as a visual analog scale displayed disordered thresholds meaning that the scoring options (0–100 mm) were not adequate. Most response options were massively underutilized. The fit of the individual items revealed that 5 out of 17 items did not fit the model.

In order to determine whether transforming the PARQ by re-categorizing of the scoring options would improve item fit and overall fit, items were re-scored. When five (0–4) scoring options were created for the items scored on a VAS, all thresholds were ordered. Response options 0 and 100 mm were kept and became 0 and 4. Response options from 1 to 29 mm became a 1 while response options 30–89 mm were scored a 2. Finally, response options 90–99 mm were scored as a 3. This rescoring structure offered the best fit for all VAS items. Once rescoring was completed, all VAS items fit the model but the five remaining items of the Morisky scale did not (Table 2). The removal of these five items resulted in the VAS items all fitting the model. The data fitted the model with an overall non-significant chi-square (95.26 *p* = 0.80).

**Table 2:** The remaining PARQ items and their psychometric properties by location order once rescored and misfitting items removed

Description/Item	Location	SE	Fit residual
Difficulty in taking prescribed medication	−1.11	0.15	−0.07
Negative reaction to taking prescribed medication	−1.98	0.10	0.54
Negative reaction to doing prescribed exercises	−1.96	0.12	0.38
Negative reaction to wearing splint	−1.73	0.24	−0.11
Difficulty in exercises	−1.72	0.13	0.56
Difficulty in wearing splint	−1.21	0.23	1.50
Adherence to taking prescribed medication	0.57	0.14	−0.39
Usefulness of taking prescribed medication	0.71	0.14	0.13
Usefulness of doing prescribed exercises	1.94	0.13	0.72
Adherence to doing prescribed exercises	2.36	0.14	−0.75
Usefulness of wearing splint	2.40	0.25	−0.33
Adherence to wearing splint	2.74	0.22	0.41

All items had non-significant chi-squares and F statistics

As for content validity, it was assessed by examination of the item-person map (Fig. 1), which shows the distributions of persons (top) and items (bottom) for the transformed PARQ. The item thresholds spread from approximately −10 to 13 logits, which is adequate.

Evidence of construct validity was ascertained as all items and persons had adequate fit statistics and none displayed DIF. The usefulness of the response categories was ascertained as all were found to have adequate fit statistics. Responses were also adequately distributed across the response categories, as there were more than 10 observations in each rating scale category.

Figure 1 is also useful for evaluating ceiling and floor effects. As no persons are located to the right or the left of the outermost items at the right and left of the graph, no ceiling or floor effects are present. The reliability index was 0.69 indicating that the transformed PARQ can reasonably discriminate persons into different ability levels. The new version of the PARQ is shown in the Appendix (https://www.dropbox.com/sh/tcdckuc6kfyd0vn/AAAu3yYHbD2uWOgCg9Lu3N3ha?dl=0).

To assess whether the transformed PARQ is unidimensional, we performed a post-hoc test of unidimensionality. According to the Smith’s t-tests [21], 6 out of 211 t-tests (2.84 %) showed significant differences in the estimates generated. Because less than 10 % of the t tests are significant, the transformed PARQ is considered unidimensional [22].

## Discussion

The aim of the present study was to evaluate the psychometric properties of the PARQ. Results of the PCA showed preliminary evidence of the multidimensionality of the original version of the PARQ and did not meet the expectations of the Rasch model. The questionnaire was transformed to determine if we could achieve a better fit to the Rasch model. These transformations include modifying the scales of some of the items (from VAS to five point Likert scale) and removing the Morisky scale items. After transformation, the items met the expectations of the Rasch model. This means that the PARQ assesses parent beliefs and behaviors related to adherence to various treatments prescribed for JIA in a cohesive manner (i.e.*,* unidimensional). This will also allow for the computation of a total score, thus facilitating the use of the questionnaire and the conduct of quantitative analysis. Such a score is important to ensure the interpretability of the measure [23] and to facilitate communication amongst researchers and health professionals.

To our knowledge, the PARQ is the first proxy-report questionnaire assessing adherence to various JIA treatments which has undergone validity testing using Rasch analysis. Furthermore, this questionnaire is the only one to evaluate adherence to JIA treatments, as well as both parents’ attitudes and beliefs about these treatments. Assessing not only adherence behaviors but also related beliefs is important to optimize adherence, as they are the most important predictors of reported adherence [2].

The Rasch-validated version of the PARQ is a first step towards establishing a total score indicator of adherence. The transformed PARQ shows preliminary evidence of unidimensionality and may allow computation of a total score. Limitations of the current study include the type of data for some of the items (ordinal) along with missing data, which precluded definite conclusions about the factor structure revealed by the PCA. Although missing data lowered the validity of the fit statistics in the Rasch analysis, the estimates of the fit statistics should not be biased [24] as the sample size was sufficient [25].

Further testing is needed to ensure the unidimensionality of the transformed version of the PARQ and to ensure its psychometric characteristics. Such endeavour is underway in a different sample of parents of youth with JIA. Using the PARQ will help to document parents’ beliefs and behaviors related to adherence to various treatments prescribed for JIA, to identify adherence issues and to put in place measures to ensure optimal adherence and health outcomes. The PARQ can play an important role in research to help assess whether adherence is optimal in intervention studies in order to judge the effectiveness of a treatment. This questionnaire can also play an important role in clinical practice to help start a discussion to better understand parents’ attitudes and beliefs about JIA treatments in order to address barriers to adherence.

Future studies will also aim at validating the child-report version (CARQ), which would allow for assessing children’s report of adherence to JIA treatments, as well as their attitudes and beliefs about these treatments. Assessing children’s and parents’ perceptions about treatments is particularly important since they have been shown to differ [5]. Using the PARQ and CARQ in combination could help health professionals to thoroughly assess family’s perceptions and behaviours about treatments, communicate information about the various treatments, and tailor treatments to both children’s and parents’ needs.

## Conclusions

The current research represents an important step in evaluating the psychometric properties of the PARQ, a questionnaire that assesses beliefs and behaviors related to adherence to treatments prescribed for JIA. The Rasch analysis led to the transformation of the PARQ showing preliminary evidence of unidimensionality. Future work will verify these findings and also validate a child-report version of this questionnaire to thoroughly assess treatment adherence and address its barriers, both in research and in clinical practice.
